# Diagnostic Model for In-Hospital Bleeding in Patients with Acute ST-Segment Elevation Myocardial Infarction: Algorithm Development and Validation

**DOI:** 10.2196/20974

**Published:** 2020-08-14

**Authors:** Yong Li

**Affiliations:** 1 Emergency and Critical Care Center Beijing Anzhen Hospital Capital Medical University Beijing China

**Keywords:** coronary disease, ST-segment elevation myocardial infarction, hemorrhage, nomogram

## Abstract

**Background:**

Bleeding complications in patients with acute ST-segment elevation myocardial infarction (STEMI) have been associated with increased risk of subsequent adverse consequences.

**Objective:**

The objective of our study was to develop and externally validate a diagnostic model of in-hospital bleeding.

**Methods:**

We performed multivariate logistic regression of a cohort for hospitalized patients with acute STEMI in the emergency department of a university hospital. Participants: The model development data set was obtained from 4262 hospitalized patients with acute STEMI from January 2002 to December 2013. A set of 6015 hospitalized patients with acute STEMI from January 2014 to August 2019 were used for external validation. We used logistic regression analysis to analyze the risk factors of in-hospital bleeding in the development data set. We developed a diagnostic model of in-hospital bleeding and constructed a nomogram. We assessed the predictive performance of the diagnostic model in the validation data sets by examining measures of discrimination, calibration, and decision curve analysis (DCA).

**Results:**

In-hospital bleeding occurred in 112 of 4262 participants (2.6%) in the development data set. The strongest predictors of in-hospital bleeding were advanced age and high Killip classification. Logistic regression analysis showed differences between the groups with and without in-hospital bleeding in age (odds ratio [OR] 1.047, 95% CI 1.029-1.066; *P*<.001), Killip III (OR 3.265, 95% CI 2.008-5.31; *P*<.001), and Killip IV (OR 5.133, 95% CI 3.196-8.242; *P*<.001). We developed a diagnostic model of in-hospital bleeding. The area under the receiver operating characteristic curve (AUC) was 0.777 (SD 0.021, 95% CI 0.73576-0.81823). We constructed a nomogram based on age and Killip classification. In-hospital bleeding occurred in 117 of 6015 participants (1.9%) in the validation data set. The AUC was 0.7234 (SD 0.0252, 95% CI 0.67392-0.77289).

**Conclusions:**

We developed and externally validated a diagnostic model of in-hospital bleeding in patients with acute STEMI. The discrimination, calibration, and DCA of the model were found to be satisfactory.

**Trial Registration:**

ChiCTR.org ChiCTR1900027578; http://www.chictr.org.cn/showprojen.aspx?proj=45926

## Introduction

Hemorrhagic complications occur in nearly 8.5% of patients with acute ST-segment elevation myocardial infarction (STEMI) during hospitalization [1,2]. Bleeding events were associated with an increased risk of adverse outcomes in patients with STEMI [3-7]. Prevention of bleeding may represent an achievable step. Mehran et al [8] developed a model to predict bleeding in patients with acute coronary syndromes; however, the model has not been validated. Alexander et al [9] developed a model to predict in-hospital major bleeding in acute myocardial infarction, but their models were only internally validated. Moa Simonsson et al [6] developed a model to predict in-hospital major bleeding in acute myocardial infarction, and the internal and temporal validity of the model was assessed. The aim of our study was to develop and externally validate a diagnostic model of in-hospital bleeding in patients with acute STEMI.

## Methods

### Statement of Ethics and Data Availability

The Ethics Committee of Beijing Anzhen Hospital Capital Medical University approved the study (approval no. 2019044X, November 18, 2019). We registered this study with the WHO International Clinical Trials Registry Platform (ICTRP) (ChiCTR.org ChiCTR1900027578, November 19, 2019).

This was a retrospective analysis, and informed consent was waived by the Ethics Committee of Beijing Anzhen Hospital Capital Medical University. All procedures performed in studies involving human participants were in accordance with the ethical standards of the institutional or national research committees and with the 1964 Helsinki Declaration and its later amendments or comparable ethical standards. The study was not conducted with animals. All data generated or analyzed during this study are included in the published paper and in Multimedia Appendix 1.

### Participant Selection

We used a Type 2b predictive model study, which is covered by a TRIPOD (Transparent Reporting of a multivariable prediction model for Individual Prognosis Or Diagnosis) statement [9]. The data were nonrandomly divided into two groups according to time: one group was used to develop a prediction model, and the other group was used for validation [9]. A Type 2b study is considered to be an external verification study [9].

The derivation cohort was 4262 hospitalized patients with acute STEMI from January 2002 to December 2013 in Beijing Anzhen Hospital, Capital Medical University. The validation cohort was 6015 hospitalized patients with acute STEMI from January 2014 to August 2019 in Beijing Anzhen Hospital, Capital Medical University. The participants were consecutively hospitalized patients with STEMI aged older than 18 years. We established the diagnosis of acute myocardial infarction (AMI) and STEMI based on the fourth universal definition of myocardial infarction [10].

### Outcomes

The outcome of interest was all-cause in-hospital bleeding not related to coronary artery bypass graft surgery or catheterization during hospitalization, as defined according to the Bleeding Academic Research Consortium criteria 2, 3, and 5 [4]. The presence or absence of in-hospital bleeding was decided blinded to the predictor variables and based on the medical record.

We selected 13 predictors according to clinical relevance and the results of baseline descriptive statistics. The potential candidate variables were age, sex, Killip classification, atrioventricular (AV) block, atrial fibrillation (AF), underwent percutaneous coronary intervention (PCI) during hospitalization, and medical history such as hypertension, diabetes, myocardial infarction, PCI, coronary artery bypass graft (CABG), cerebrovascular disease, and chronic kidney disease (CKD). All these variables were determined based on the patients’ medical records. AF was defined as all types of AF during hospitalization. AV block was defined as all types of AV block during hospitalization.

Our numbers of samples and events exceeded the minima required for all approaches; each candidate variable included at least 10 events for model derivation and at least 100 events for validation studies [9].

We excluded patients who lacked information on the key predictors of age and Killip classification. The reason for exclusion of all patients was lack of Killip classification.

We maintained all continuous data as continuous and retained the original scale. Based on the significant variables generated by univariate logistic regression, we constructed a multivariate logistic regression model using the backward variable selection method. We used the Akanke information criterion (AIC) and Bayesian information criterion (BIC) to select predictors. These criteria considered the model fitting and penalized the estimated number of parameters, which was equivalent to using α=.157 [9].

We assessed the predictive performance of the diagnostic model in the validation data set by examining measures of discrimination, calibration, and decision curve analysis (DCA) [9,11].

Discrimination was defined as the ability of the diagnostic model to differentiate between patients with and without in-hospital bleeding. This measure was quantiﬁed by calculating the area under the receiver operating characteristic (ROC) curve (AUC) [9].

Calibration referred to how closely the predicted in-hospital bleeding agrees with the observed in-hospital bleeding [9]. The Brier score is an aggregate measure of disagreement between the observed outcome and a prediction based on the average squared error difference.

We used DCA to describe and compare the clinical effects of the diagnostic model [9].

We performed statistical analyses with STATA version 15.1 (StataCorp), R version 4.0.0 (R Project), and the RMS package developed by Harrell et al [12].

## Results

The study was approved by the ethics committee on November 18, 2019. Data collection started on November 26, 2019. As of submission of the manuscript, 10,277 people had been recruited for the study.

**Figure 1:**
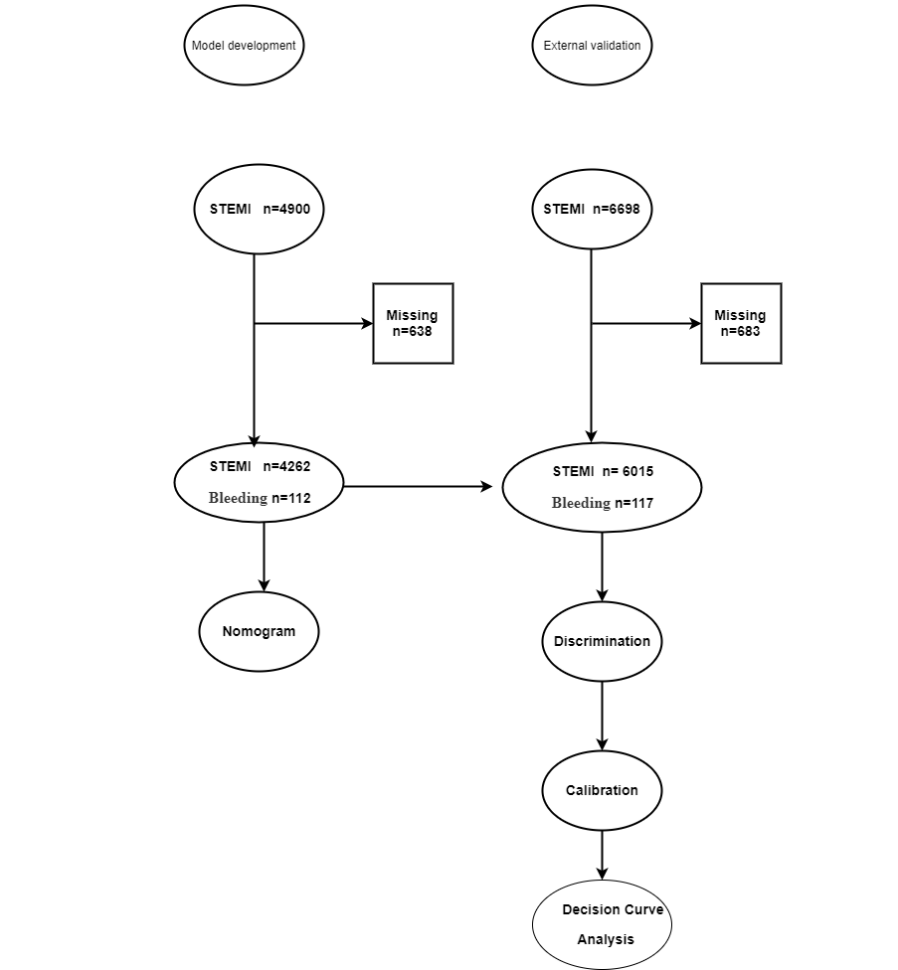
Flow diagram of the study. STEMI: ST-segment elevation myocardial infarction.

A flow diagram of the study is presented in Figure 1.

In the development data set, 112 of 4262 hospitalized patients (2.6%) experienced in-hospital bleeding. The patients’ baseline characteristics are shown in Table 1. Nine variables (age, sex, Killip classification, AVB, AF, history of CABG, history of diabetes, history of CKD, and underwent PCI during hospitalization) were significantly different in the two groups of patients (α=.157).

**Table 1:** Demographic and clinical characteristics of patients with and without in-hospital bleeding in the development data set (N=4262).

Characteristic		Total (N=4262)	In-hospital bleeding (n=112)	No bleeding (n=4150)	*P* value
Age (years, range 21-99), mean (SD)		60 (13)	70 (10)	60 (13)	<.001
Male sex, n (%)		3248 (76.2)	73 (65.2)	3175 (76.5)	.006
**Medical history, n (%)**					
	Hypertension	2372 (55.7)	63 (56.3)	2309 (55.6)	.90
	Diabetes	1246 (29.2)	42 (37.5)	1204 (29.0)	.053
	Myocardial infarction	426 (10.0)	13 (11.6)	413 (10.0)	.57
	PCI^a^	228 (5.3)	7 (6.3)	221 (5.3)	.67
	CABG^b^	28 (0.7)	2 (1.8)	26 (0.6)	.15
	CKD^c^	95 (2.2)	7 (6.3)	88 (2.1)	.006
	HCD^d^	338 (7.9)	11 (9.8)	327 (7.9)	.45
**Killip classification,** **n (%)**					
	I	769 (18)	13 (11.6)	756 (18.2)	.08
	II	2565 (60.2)	31 (27.7)	2534 (61.1)	<.001
	III	533 (12.5)	32 (28.6)	501 (12.1)	<.001
	IV	395 (9.3)	36 (32.1)	359 (8.7)	<.001
AF^e^		243 (5.7)	15 (13.4)	228 (5.5)	.001
AVB^f^		197 (4.6)	13 (11.6)	184 (4.4)	.001
Underwent PCI		3103 (72.8)	50 (44.6)	3053 (73.6)	<.001

^a^PCI: percutaneous coronary intervention.

^b^CABG: coronary artery bypass graft.

^c^CKD: chronic kidney disease.

^d^HCD: history of cerebrovascular disease.

^e^AF: atrial fibrillation.

^f^AVB: atrioventricular block.

After application of the backward variable selection method, AIC, and BIC, age remained a significant independent predictor of in-hospital bleeding; Killip classification remained a rank variable of in-hospital bleeding. These results are shown in Table 2 and Table 3.

**Table 2:** Predictors of in-hospital bleeding obtained from multivariable logistic regression models (odds ratio) in the development data set.

In-hospital bleeding	Odds ratio	Standard error	Z	Pr>| Z |	95% CI
Age	1.047443	0.0095986	5.06	<.001	1.028798-1.066426
Killip III	3.265072	0.8100203	4.77	<.001	2.007804-5.309632
Killip IV	5.132613	1.240357	6.77	<.001	3.196212-8.242169
Constant	0.0007621	0.0004685	–11.68	<.001	0.0002285-0.0025424

**Table 3:** Predictor of in-hospital bleeding obtained from multivariable logistic regression models (coefficients) in the development data set.

In-hospital bleeding	Coefficient	Standard error	Z	Pr>| Z |	95% CI
Age	0.0463523	0.0091638	5.06	<.001	0.0283915 to 0.0643131
Killip III	1.183282	0.2480865	4.77	<.001	0.6970414 to 1.669523
Killip IV	1.635615	0.2416619	6.77	<.001	1.161966 to 2.109263
Constant	–7.179377	0.6146614	–11.68	<.001	–8.384092 to –5.974663

**Figure 2:**
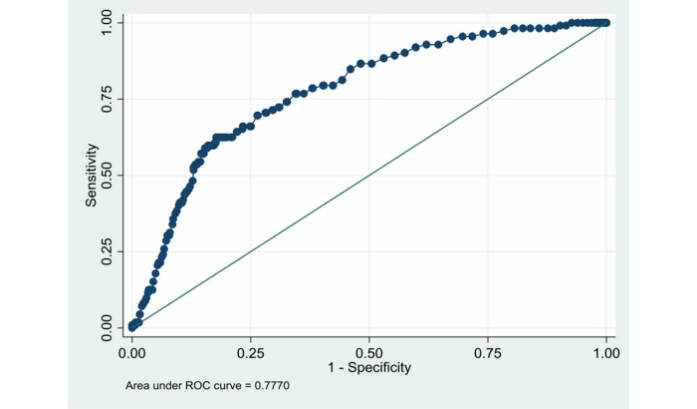
ROC curve for the identification of patients with in-hospital bleeding in the development dataset. ROC: receiver operating characteristic.

According to the above risk factors, we can calculate the predicted probability of in-hospital bleeding using the formula *P*= 1/(1 + exp(–(–7.179377 + 0.0463523 × AGE(years) + 1.183282 × KIII + 1.635615 × KIV))), where KIII is Killip III (0 = No, 1 = Yes) and KIV is Killip IV (0 = No, 1 = Yes). The ROC curve was drawn (Figure 2). The AUC was 0.777 (SD 0.021, 95% CI 0.73576-0.81823).

**Figure 3:**
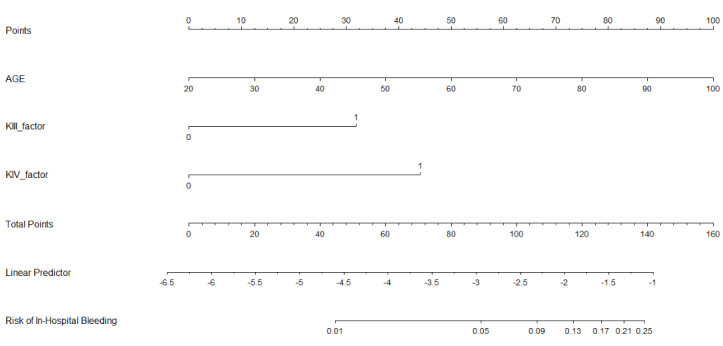
Nomogram for predicting in-hospital bleeding in patients with acute ST-segment elevation myocardial infarction. AGE: age (years); KIII-factor: Killip III; KIV-factor: Killip IV.

We constructed the nomogram (Figure 3) using the development database based on an independent prognostic marker (age) and a rank variable (Killip classification). To use the nomogram, the patient’s age is found on the AGE axis, and a straight line is then drawn upward to the Points axis to determine how many points toward progression the patient receives for their age. The steps are repeated for the other axes, with a straight line drawn upward each time toward the points axis. The points received for each predictor are summed, and the sum is found on the total points axis. A straight line is drawn down to the Risk of In-Hospital Bleeding axis to find the patient’s probability of in-hospital bleeding.

A total of 117 of 6015 hospitalized patients in the validation data set (1.9%) suffered in-hospital bleeding. The baseline characteristics of the patients are shown in Table 4. We can calculate the predicted probability of in-hospital bleeding using the formula *P*= 1/(1 + exp(–(–7.179377 + .0463523 × age (years) + 1.183282 × KIII + 1.635615 × KIV))), where KIII is Killip III (0 = No, 1 = Yes) and KIV is Killip IV (0 = No, 1 = Yes).

**Figure 4:**
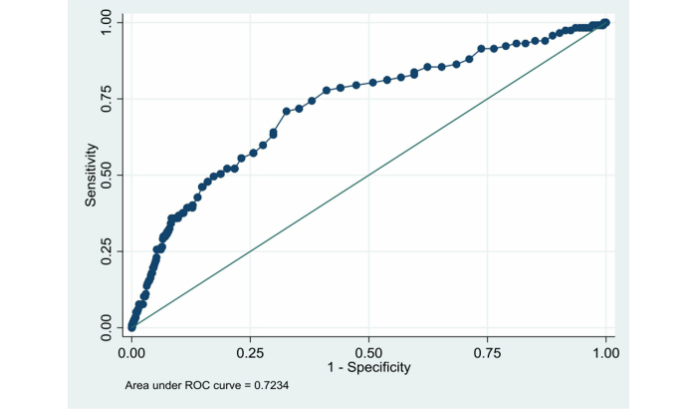
ROC curve for the identification of patients with in-hospital bleeding in the validation data set. ROC: receiver operating characteristic.

We drew the ROC curve (Figure 4). The AUC was 0.7234 (SD 0.0252, 95% CI 0.67392-0.77289).

**Figure 5:**
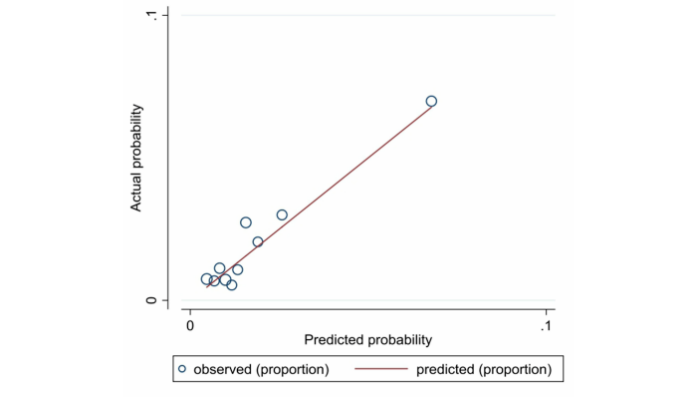
Calibration plot with distribution of the predicted probabilities for individuals with and without in-hospital bleeding in the validation data set.

We drew a calibration plot (Figure 5) with the distribution of the predicted probabilities for individuals with and without in-hospital bleeding in the validation data set. The Hosmer-Lemeshow χ^2^_10_ value was 10.64, Pr>χ^2^ was 0.3859>.05, and the Brier score was .0188 (<.25).

**Figure 6:**
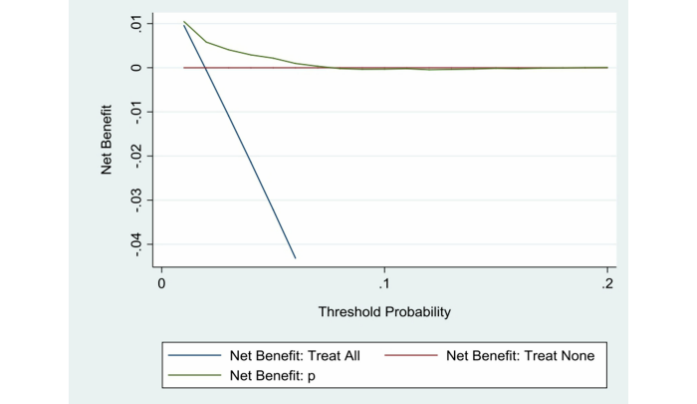
Decision curve analysis of the validation data set.

Figure 6 shows the DCA of the validation data set.

**Table 4:** Demographic and clinical characteristics of patients with and without in-hospital bleeding in the validation data set (N=6015).

Characteristic		Total (N=6015)	In-hospital bleeding (n=117)	No bleeding (n=5898)	*P* value
Age (years, range 21-92), mean (SD)		59 (12)	64 (12)	58 (12)	<.001
Male sex, n (%)		4894 (81.4)	86 (73.5)	4808 (81.5)	.03
**Medical history, n (%)**					
	Hypertension	3427 (57.0)	65 (55.6)	3362 (57)	.75
	Diabetes	1822 (30.3)	40 (34.2)	1782 (30.2)	.36
	Myocardial infarction	433 (7.2)	14 (12)	419 (7.1)	.047
	PCI^a^	575 (9.6)	18 (15.4)	557 (9.4)	.03
	CABG^b^	51 (0.8)	3 (2.6)	48 (0.8)	.05
	CKD^c^	145 (2.4)	4 (3.4)	141 (2.4)	.48
	HCD^d^	421 (7.0)	12 (10.3)	409 (6.9)	.17
**Killip classification,** **n (%)**					
	I	4234 (70.4)	45 (38.5)	4189 (71.0)	<.001
	II	1188 (19.7)	31 (26.5)	1157 (19.6)	.07
	III	266 (4.4)	11 (9.4)	255 (4.3)	.01
	IV	330 (5.5)	30 (25.6)	300 (5.1)	<.001
AF^e^, n (%)		275 (4.6)	12 (10.3)	263 (4.5)	.004
AVB^f^, n (%)		119 (2.0)	3 (2.6)	116 (2.0)	.65
Underwent PCI n (%)		4564 (75.9)	70 (59.8)	4494 (76.2)	<.001

^a^PCI: percutaneous coronary intervention.

^b^CABG: coronary artery bypass grafting.

^c^CKD: chronic kidney disease.

^d^HCD: cerebrovascular disease.

^e^AF: atrial fibrillation.

^f^AVB: atrioventricular block.

## Discussion

### Principal Findings

In our study, advanced age and high Killip classification were associated with increased risk of in-hospital bleeding in patients with acute STEMI. The formula or nomogram could be used to predict in-hospital bleeding. Specific strategies should be used to reduce the risk of in-hospital bleeding, such as ensuring the appropriate dose of antithrombotic drugs.

The predictive performance of the diagnostic model in the validation data set was assessed by examining measures of discrimination, calibration, and DCA. The AUC was 0.7234 (SD 0.0252, 95% CI 0.67392-0.77289) in the validation data set. The Hosmer-Lemeshow χ^2^_10_ value was10.64, Pr>χ^2^ was 0.3859>.05. and the Brier score was <.25. The discrimination, calibration, and DCA results were satisfactory.

A high Killip classification has been associated with increased risk of bleeding [3,7,13]. In our study, patients with Killip class IV were at 5.1 times higher risk of in-hospital bleeding than patients with Killip classes I to III. Insufficient tissue perfusion adversely affected the coagulation system and platelet function [13]. Insufficient tissue perfusion may cause gastritis or ulceration and increase the possibility of gastrointestinal bleeding [13].

Advanced age has been reported to be an independent risk factor of bleeding [3,13-17]. Age may change the balance between the risks and benefits of treatment strategies [18]. The cause of the higher risk of bleeding in older people may be multifactorial, including decreased kidney function and increased sensitivity to anticoagulants [19]. It has been speculated that the presence of local vascular changes is an explanation for the increased incidence of bleeding complications in older patients [20]. Stomach protection is recommended for older patients [21].

Moscucci et al [20] observed that older age, female sex, history of bleeding, and renal insufficiency were independent predictors of major bleeding among 8151 patients with STEMI, 7440 patients with non–ST-segment elevation myocardial infarction (NSTEMI), and 8454 patients with unstable angina registered in the Global Acute Coronary Events Registry (GRACE). Spencer et al [22] found that major bleeding occurred in 2.8% of 40,087 patients with AMI enrolled in the GRACE. These patients were older, more severely ill, and more likely to undergo invasive procedures. Subherwal et al [23] used 71,277 patients to derive and 17,857 patients to validate a model to stratify the risk of major bleeding in patients with NSTEMI. This was a form of internal validation, as their development and validation cohorts were created randomly rather than nonrandomly [9]. Nikolsky et al [19] found 7 independent predictors of major bleeding after PCI using the femoral approach, and the AUC was 0.62 in the validation data set.

Roxana Mehran et al [8] used 17,421 patients to derive a model that identifies 6 independent baseline predictors to predict bleeding in patients with acute coronary syndromes; however, this model has not been validated. KP Alexander et al [24] used 72,313 patients to develop and 17,960 patients to validate a model to predict in-hospital major bleeding during myocardial infarction care. This was also a form of internal validation because their cohorts were randomly created [9]. Moa Simonsson et al [6] used 97,597 patients to develop a model to predict in-hospital major bleeding in acute myocardial infarction. The internal and temporal validity of the model were assessed; the temporal validity of the score was assessed using internal-external cross-validation [6].

Our diagnostic model of in-hospital bleeding builds upon these studies in several ways. Our model was externally validated. It provides an absolute value rather than a relative value. It includes only two baseline factors, namely age and Killip classification. It can be easily calculated at patient presentation. It can remain discriminatory irrespective of which treatment was used (eg, invasive care or antithrombotic drugs), thereby improving its effectiveness in clinical decision-making. It was developed using unselected real-world populations, including patients who underwent initial invasive strategies and revascularization as well as patients who were conservatively treated without catheterization. Algorithms that can help physicians evaluate diagnoses should be simple and easy to apply, and they should use clinical data that is routinely provided by the hospital. The nomogram we constructed for in-hospital bleeding captures most of the diagnostic information provided by the complete logistic regression model and is easy to use.

### Limitations

The present analysis has a few limitations. This was a single-center study. Some patients were selected >10 years ago; therefore, their treatment may not represent current standards and techniques. We did not include bleeding related to catheterization. The use of antithrombotic drugs and previous bleeding history were not obtained in this study; therefore, we could not determine the impact of anticoagulation or previous bleeding history on bleeding risk. Finally, the C statistics of the in-hospital bleeding model in the study were modest (0.777 in the derivation cohort and 0.7234 in the validation cohort).

### Conclusion

We developed and externally validated a diagnostic model of in-hospital bleeding in patients with acute STEMI.

## Appendix

Multimedia Appendix 1 Supplementary materials. The data are the demographic and clinical characteristics of hospitalized patients with acute STEMI. AGE: age; ALLAF: atrial fibrillation; AVB: atrioventricular block; BLOOD: all-cause bleeding; CABG: history of coronary artery bypass graft; CKD: history of chronic kidney disease; DM: history of diabetes; HBP: history of hypertension; HCD: history of cerebrovascular disease; HPCI: history of percutaneous coronary intervention; KI: Killip I; KII: Killip II; KIII: Killip III; KIV: Killip IV; OMI: history of myocardial infarction; PCI: underwent PCI during hospitalization; S: sex.

## Abbreviations

AF: atrial fibrillation
AIC: Akanke information criterion
AMI: acute myocardial infarction
AUC: area under the receiver operating characteristic curve
AV: atrioventricular
BIC: Bayesian information criterion
CABG: coronary artery bypass graft
CKD: chronic kidney disease
DCA: decision curve analysis
HCD: history of cerebrovascular disease
GRACE: Global Registry of Acute Coronary Events
MI: myocardial infarction
NSTEMI: non–ST-segment elevation myocardial infarction
PCI: percutaneous coronary intervention
ROC: receiver operating characteristic
STEMI: ST-segment elevation myocardial infarction
TIMI: thrombolysis in myocardial infarction
TRIPOD: Transparent Reporting of a multivariable prediction model for Individual Prognosis Or Diagnosis
